# Biochemical role of homocysteine in immune modulation and cytokine dynamics in acute ischemic stroke: Implications for stroke-associated infections

**DOI:** 10.5937/jomb0-56695

**Published:** 2025-09-05

**Authors:** Jiayi Cai, Qiuxin Xu, Haofeng Shen

**Affiliations:** 1 Shanghai Putuo District Central Hospital, Department of Rehabilitation, Shanghai, China; 2 Shanghai Shengxi Rehabilitation Center, Shanghai, China

**Keywords:** ischemic stroke, homocysteine, cytokine dynamics, immune modulation, stroke-associated infection, inflammatory pathways, ishemijski moždani udar, homocistein, dinamika citokina, imunomodulacija, infekcija povezana sa moždanim udarom, inflamatorni putevi

## Abstract

**Background:**

Ischemic stroke is a leading cause of morbidity and mortality, with immune dysregulation contributing to its progression. Elevated homocysteine (Hcy) levels are implicated in altering immune responses and increasing stroke severity. This study aimed to investigate the biochemical role of serum homocysteine in modulating immune responses, particularly cytokine profiles, and its association with post-stroke infections in patients with acute ischemic stroke.

**Methods:**

A cohort of 106 patients with acute ischemic stroke was divided into Low-, Medium-, and High-Hcy groups. Serum levels of cytokines (IL-6, IL-4, IFN-g, IL-10) and immune modulation markers (e.g., IFN-g/IL-4 ratio) were quantified. The presence of stroke-associated infections (SAI) was recorded, and its relationship with immune parameters was analyzed.

**Results:**

The High-Hcy group showed significantly higher serum levels of IL-6, IFN-g, and IL-10 compared to the Low-Hcy group (P < 0.05), suggesting a pro-inflammatory bias. In patients with SAI, IL-4 levels were notably elevated, and the IFN-g/IL-4 ratio indicated an immune suppressive trend. Although stroke severity was similar across groups, those with heightened immune dysregulation were more prone to infections.

**Conclusions:**

Elevated homocysteine levels induce a shift in immune responses, emphasizing the dual role of cytokines in stroke pathophysiology. Targeting these biochemical pathways may present novel therapeutic strategies to mitigate stroke complications.

## Abstract (sr)

**Uvod:**

Ishemijski moždani udar je vodeći uzrok morbiditeta i mortaliteta, a imunološka disregulacija doprinosi njegovom napredovanju. Povišeni nivoi homocisteina (Hci) su uključeni u promenu imunoloških odgovora i povećanje težine moždanog udara. Ova studija je imala za cilj da istraži biohemijsku ulogu serumskog homocisteina u modulaciji imunoloških odgovora, posebno profila citokina, i njegovu povezanost sa infekcijama nakon moždanog udara kod pacijenata sa akutnim ishemijskim moždanim udarom.

**Metode:**

Kohorta od 106 pacijenata sa akutnim ishemijskim moždanim udarom podeljena je u grupe sa niskim, srednjim i visokim Hci. Nivoi citokina u serumu (IL-6, IL-4, IFN-g, IL-10) i markera imunomodulacije (npr. odnos IFN-g/IL-4) su kvantifikovani. Zabeleženo je prisustvo infekcija povezanih sa moždanim udarom (SAI) i analiziran je njihov odnos sa imunološkim parametrima.

**Rezultati:**

Grupa sa visokim nivoom Hci pokazala je značajno više nivoe IL-6, IFN-g i IL-10 u serumu u poređenju sa grupom sa niskim Hci (P < 0,05), što ukazuje na proinflamatornu pristrasnost. Kod pacijenata sa SAI, nivoi IL-4 su bili značajno povišeni, a odnos IFN-g/IL-4 je ukazivao na imunosupresivni trend. Iako je težina moždanog udara bila slična u svim grupama, oni sa povećanom imunološkom disregulacijom bili su skloniji infekcijama.

**Zaključak:**

Povišeni nivoi homocisteina izazivaju promenu u imunološkim odgovornim infekcijama t-moždanog udara, naglašavajući dvostruku ulogu citokintofiziologije. Usmeravanje na ove biohemijske puteve može predstavljati nove terapeutske strategije za ublažavanje komplikacija moždanog udara.

## Introduction

According to the World Health Organization (WHO), stroke is the second leading cause of death globally, with ischemic stroke accounting for 87% of all cases. Its high rate of disability imposes a significant social and economic burden [1]. The immune system plays a pivotal role in the pathophysiology of ischemic stroke, interacting with disease progression and offering potential therapeutic targets [2]. As a result, the immunological mechanisms underlying stroke have garnered increasing research attention.

The pathological basis of ischemic stroke is largely attributed to atherosclerosis (AS), a chronic inflammatory condition of the vascular wall characterized by endothelial injury, lipid deposition and oxidation, and immune cell infiltration. Immune cells, including lymphocytes and macrophages, contribute to plaque formation and the destabilization of atherosclerotic plaques, which are key triggers of ischemic stroke [3] [4]. Macrophages polarize into pro-inflammatory M1 and anti-inflammatory M2 subtypes. M1 macrophages exacerbate inflammation and promote plaque instability through cytokines such as IL-1β and IL-12, whereas M2 macrophages secrete anti-inflammatory mediators like IL-10 and TGF-β, facilitating tissue repair [5]. Similarly, Th1 cells release IFN-γ, enhancing inflammation, while Treg cells suppress inflammatory responses and stabilize plaques. These insights highlight immune processes as promising therapeutic targets for AS and stroke.

In ischemic stroke, the immune system exhibits dual roles, influencing both acute and long-term outcomes. Following stroke onset, astrocytes and microglia become activated, releasing inflammatory mediators and attracting peripheral immune cells to the ischemic site. While these responses aggravate tissue damage, M2 microglia and Treg cells contribute to inflammation resolution and repair. During the chronic recovery phase, immune status influences neurogenesis, angiogenesis, and autoimmunity, affecting long-term functional recovery [6].

Systemically, stroke triggers a biphasic immune response: an initial pro-inflammatory phase characterized by elevated IL-6 and IFN-γ, followed by immunosuppression marked by reduced lymphocyte counts, Th1/Th2 polarization, and spleen atrophy [7] [8]. This immunosuppressive state increases susceptibility to infections such as pneumonia, a major contributor to post-stroke mortality. Elevated neutrophilto-lymphocyte ratio (NLR), a biomarker of immune dysregulation, strongly correlates with severe infections, worse outcomes, and higher mortality. Sympathetic activation and glucocorticoid release further exacerbate these changes by suppressing IFN-γ production and promoting IL-10 secretion.

Additionally, elevated serum homocysteine (Hcy) is an independent risk factor for cerebrovascular diseases and mortality [9] [10]. High Hcy levels promote T-cell differentiation into pro-inflammatory Th1 and Th17 subtypes, elevating cytokines like IL-2, IFN-γ, and TNF-α, thereby exacerbating inflammation and plaque progression. Recent findings suggest that Hcy-induced IL-17A expression involves the p38 MAPK pathway and NSun2-mediated mRNA methylation. These mechanisms underline Hcy’s role in immune dysregulation and its association with poor stroke outcomes, including severe neurological deficits and high recurrence risk [11].

This study aimed to analyze the factors influencing the immune status of ischemic stroke patients, providing insights into the interplay between immune mechanisms and stroke pathophysiology, progression, prognosis, and recovery. Understanding these relationships could pave the way for novel immunomodulatory interventions to improve patient outcomes.

## Materials and methods

### Enrolled participants

A total of 106 patients with acute ischemic stroke hospitalized in the Department of Neurology of our hospital were enrolled in this study.

The inclusion criteria were as follows: onset of symptoms within 72 hours prior to enrollment, a National Institutes of Health Stroke Scale (NIHSS) score [12] between 4 and 13, age between 18 and 80 years, and voluntary participation in the study, regardless of gender.

The exclusion Criteria were as follows: Patients meeting any of the following criteria were excluded from the study: (1) presence of other neurological diseases such as intracranial arterial dissection, aneurysm, vasculitis, or vascular malformations; (2) prior thrombolytic or thrombectomy treatment; (3) confirmed or suspected cerebral embolism; (4) abnormal liver or kidney function, defined as aspartate aminotransferase (AST) or alanine aminotransferase (ALT) levels exceeding three times the upper normal limit, or creatinine clearance <0.6 mL/s or serum creatinine levels >265 mmol/L; (5) presence of autoimmune diseases, history of immunotherapy, or malignancy; (6) recent history of severe trauma or major surgery.

### Grouping based on homocysteine levels

Participants were categorized into three groups based on their serum homocysteine (Hcy) levels: the Low-Hcy Group (Hcy ≤ 12.3 mmol/L, n = 33), the Medium-Hcy Group (12.3 mmol/L < Hcy ≤ 17.4 mmol/L, n = 36), and the High-Hcy Group (Hcy > 17.4 mmol/L, n = 37). The classification was determined by the tertile distribution of Hcy levels among all participants.

### Grouping based on stroke-associated infection

Participants were further divided into two groups based on the presence of stroke-associated infection (SAI): the SAI group (n = 35) and the non-SAI (nSAI) group (n = 71). SAI was defined by the presence of both (1) clinical signs of infection, such as urinary symptoms, cough, sputum production, or fever, and (2) laboratory or imaging evidence of infection, such as elevated white blood cell count (>10 × 10 /L), increased urinary white blood cell count, or pneumonia observed on chest CT scans. The nSAI group was defined by the absence of these clinical and laboratory findings. Infections were diagnosed within the first 7 days post-stroke and were confirmed during hospitalization.

### Sample collection

Peripheral blood samples (2–3 mL) were collected immediately upon patient admission using yellowtopped serum-separation tubes. After centrifugation at 4°C, 3000 r/min for 5 minutes, the supernatant was separated and stored at -80°C for subsequent analysis.

### Detection of cytokines

Standard cytokine solutions were prepared by diluting cytokine standards in 2 mL of diluent, ensuring minimal disturbance by avoiding vortexing and gently mixing by pipetting. The solution was allowed to equilibrate at room temperature for 15 minutes and was designated as the »Top standard.« To create the serial dilution curve, ten flow cytometry tubes were labeled as follows: blank tube, Top standard, 1/2 standard, 1/4 standard, 1/8 standard, 1/16 standard, 1/32 standard, 1/64 standard, 1/128 standard, 1/256 standard, and 1/512 standard. To each tube, 300 μL of diluent was added, except for the Top standard tube. Subsequently, 300 mL of the Top standard was transferred to the 1/2 standard tube and mixed gently, and this serial dilution process was repeated for all subsequent tubes, ensuring consistent and uniform mixing at each dilution step.

Each bead type was vortexed for at least 15 seconds prior to use to ensure uniform distribution and optimal biochemical reactivity. The required volume of each bead type was calculated based on the total number of tubes (including standards, samples, and blank tubes) and was set at 10 μL per tube. These calculated volumes were combined into a single centrifuge tube, vortexed thoroughly, and designated as the »mixed bead suspension.« The mixture was centrifuged at 200 × g for 5 minutes, and the supernatant was discarded. The beads were then resuspended in Soluble Protein Master Buffer at a volume of 50 μL per tube. The suspension was incubated at room temperature, shielded from light, for 30 minutes to ensure optimal bead activation.

Subsequently, 50 μL of the mixed bead suspension was added to each tube, followed by the addition of 50 μL of the corresponding standard, sample, or diluent (for the blank tube). To facilitate specific cytokine binding, 50 μL of antibody solution was added to each tube. The tubes were incubated at room temperature, protected from light, for 3 hours, allowing sufficient time for antigen-antibody interactions. After the incubation, 1 mL of wash buffer was added to each tube, and the tubes were centrifuged at 200 × g for 5 minutes. The supernatant was discarded, and the beads were resuspended in 300 μL of wash buffer to remove unbound material.

Finally, the samples were analyzed using a BD FACS Calibur flow cytometer (BD Biosciences, Franklin Lakes, NJ, USA), which enables the precise quantification of cytokine levels based on the fluorescence intensity of the beads, thus providing accurate biochemical measurements of the cytokine profile in the samples.

### Collection of clinical information

General patient data, including age, sex, history of hypertension, diabetes, and smoking, were recorded. Laboratory parameters, such as peripheral blood white blood cell count, absolute and relative neutrophil and lymphocyte counts, low-density lipoprotein cholesterol, homocysteine, uric acid, and fasting blood glucose levels, were measured. Imaging data, including infarct size measured by MRI and diffusionweighted imaging (DWI), were collected. Neurological function was evaluated using the NIHSS score on the day of admission. Infection status was determined based on the presence of clinical symptoms (e.g., urinary symptoms, cough, fever) and laboratory or imaging findings (e.g., elevated white blood cell count, increased urinary white blood cell count, pneumonia on chest CT).

### Statistical analysis

Statistical analyses were conducted using Statistic Package for Social Science (SPSS) 25.0 (IBM, Armonk, NY, USA). Categorical data were expressed as counts and percentages, while continuous variables were presented as mean ± standard deviation (for normal distributions) or median and interquartile range (for non-normal distributions). The chi-square test was used for categorical variables. For continuous variables, the t-test was applied to normally distributed data, and the Mann-Whitney U test was used for non-normal data. ANOVA with LSD-t tests or the Kruskal-Wallis H test was employed for comparisons among three groups. Statistical significance was defined as P < 0.05.

## Results

### Impact of homocysteine on the cytokine levels of acute ischemic stroke patients

The enrolled 106 patients with acute ischemic stroke were categorized into Low-Hcy, Medium-Hcy, and High-Hcy groups based on tertiles of serum homocysteine levels. No significant differences were observed in baseline characteristics, including gender, age, hypertension, diabetes, smoking history, lipid levels, fasting glucose, uric acid levels, or NIHSS scores among the groups (Table 1).

**Table 1:** Baseline Characteristics of Patients by Homocysteine Level. IQR: interquartile

Variable	Low-Hcy Group <br>(n=33)	Medium-Hcy Group <br>(n=36)	High-Hcy Group <br>(n=37)	P Value
Male (%)	23 (69.7%)	25 (69.4%)	26 (70.3%)	0.890
Female (%)	10 (30.3%)	11 (30.6%)	11 (29.7%)	0.873
Hypertension (%)	18 (54.5%)	24 (66.7%)	22 (59.5%)	0.818
Diabetes (%)	13 (39.4%)	9 (25.0%)	13 (35.1%)	0.607
Smoking (%)	17 (51.5%)	29 (80.6%)	22 (59.5%)	0.431
Age (years)	58.4 (34–74)	60.7 (35–75)	61.2 (39–76)	0.703
Uric Acid (median, IQR) (μmol/L)	3.07±0.69	2.82±0.782	2.85±0.73	0.549
Fasting glucose (median, IQR)	297.52 (276.3–335.1)	319.1 (283.5–377.7)	296.8 (286.8–398.2)	0.803
Fasting glucose (mmol/L)	6.12 (4.73–9.02)	5.4 (4.78–5.99)	5.61 (5.21–8.18)	0.901
NIHSS Score	7.5 (7–9)	8.5 (5–11)	9.5 (6–11)	0.487

Analysis of cytokine levels among the groups revealed the following results: (1) Serum levels of IFN-γ, IL-6, and IL-10 were significantly higher in the High-Hcy Group compared to the Low-Hcy Group (P < 0.05). IL-2 showed an increasing trend but was not statistically significant. (2) Serum IL-6 levels were significantly elevated in the Medium-Hcy Group compared to the Low-Hcy Group. Due to the significant differences observed in IL-6, IFN-γ, and IL-10 levels among the groups, pairwise comparisons were conducted. The results showed significant differences in IL-6 levels between the Low-Hcy and Medium-Hcy Groups, as well as between the Low-Hcy and High- Hcy Groups. Median and mean ranks for IL-6 were significantly higher in the Medium-Hcy and High-Hcy Groups compared to the Low-Hcy Group. Additionally, IFN-γ levels were significantly different between the Low-Hcy and High-Hcy Groups, with the median and mean ranks for IFN-γ being significantly higher in the High-Hcy Group. Similarly, IL-10 levels in the High-Hcy Group were significantly higher than those in the Low-Hcy Group, with statistically significant differences in median and mean ranks (Table 2).

**Table 2:** Cytokine Levels Among Homocysteine Groups.

Cytokine	Low-Hcy Group	Medium-Hcy Group	High-Hcy Group	P Value
IL–6 (pg/mL)	2.85 (1.86–5.90)	8.64 (4.59–30.31)	9.66 (5.15–35.64)	0.012
IL-4 (pg/mL)	0.48 (0.22–0.83)	0.57 (0.27–0.76)	0.70 (0.46–1.21)	0.194
IL-2 (pg/mL)	0.62 (0.34–0.72)	0.47 (0.37–0.98)	0.68 (0.38–2.3)	0.503
IL–17A (pg/mL)	3.91 (1.85–4.36)	2.37 (1.30–4.56)	3.10 (1.15–4.72)	0.705
IFN-γ (pg/mL)	0.30 (0.01–0.61)	0.53 (0.18–1.13)	1.01 (0.51–1.95)	0.009
TNF-α (pg/mL)	1.34 (0.62–2.04)	1.42 (0.55–2.85)	1.75 (0.43–2.76)	0.769
IL-10 (pg/mL)	0.83 (0.33–1.35)	1.24 (0.76–2.70)	1.39 (1.00–3.78)	0.027

### Impact of homocysteine on the immune status of acute ischemic stroke patients

Analysis of immune status indicators, including the IFN-γ/IL-4 ratio, TNF-α/IL-4 ratio, and TNF-α/IL- 10 ratio, revealed an increasing trend in the IFN-γ/IL- 4 ratio with higher homocysteine levels; however, the differences were not statistically significant (Table 3).

**Table 3:** Immune Status Indicators Among Homocysteine Groups.

Immune Status Indicator	Low-Hcy Group	Medium-Hcy Group	High-Hcy Group	P Value
TNF-α/IL-4	2.15 (1.60–4.15)	2.36 (0.62–12.21)	1.33 (1.00–3.39)	0.737
TNF-α/IL-10	2.08 (0.60–3.4)	0.92 (0.23–3.80)	0.63 (0.27–1.87)	0.492
IFN-γ/IL-4	0.62 (0.05–1.28)	1.15 (0.39–2.59)	3.11 (0.19–3.14)	0.296

### Immune status and post-stroke infections in acute ischemic stroke patients

Based on the presence or absence of post-stroke infections, a total of 35 patients were categorized into Stroke-Associated Infection (SAI) group, the rest 71 were categorized into the non-Stroke-Associated Infection (nSAI) groups Baseline characteristics of the two groups showed no significant differences (P > 0.05, Table 4).

**Table 4:** Comparison of Baseline Characteristics Between SAI and nSAI Groups.

Variable	SAI Group <br>(n=35)	nSAI Group <br>(n=71)	P Value
Male (%)	24 (68.6%)	48 (67.6%)	0.459
Female (%)	11 (31.4%)	23 (32.4%)	0.742
Hypertension <br>(%)	27 (77.1%)	37 (52.1%)	0.087
Diabetes (%)	15 (42.9%)	14 (19.7%)	0.241
Smoking (%)	26 (74.3%)	40 (56.3%)	0.403
Age (years)	56 (34–75)	59 (37–74)	0.702
LDL-C <br>(mmol/L)	2.45±0.65	2.97±0.71	0.103
Uric Acid <br>(μmol/L)	364.69 <br>(264.73–358.36)	327.19 <br>(288.92–381.12)	0.806
Homocysteine <br>(mmol/L)	15.21 <br>(11.31–21.59)	12.59 <br>(11.17–19.23)	0.307
NIHSS Score	8.5 (7–10)	8.4 (5–11)	0.903

Comparison of cytokine levels between the two groups revealed that IL-4 levels were significantly higher in the SAI group (P < 0.001), while other cytokines showed no significant differences (Table 5).

**Table 5:** Comparison of Cytokine Levels Between SAI and nSAI Groups.

Cytokine	SAI Group	nSAI Group	P Value
IL-6 <br>(pg/mL)	7.19 (4.6–25.42)	6.09 (2.0–20.01)	0.498
IL-4 <br>(pg/mL)	0.74 (0.61–1.31)	0.49 (0.24–0.68)	<0.001
IL-2 <br>(pg/mL)	0.99 (0.38–4.42)	0.44 (0.35–0.75)	0.129
IL-17A <br>(pg/mL)	3.25 (1.82–6.38)	3.12 (1.43–4.04)	0.394
IFN-γ <br>(pg/mL)	0.6 (0.18–1.81)	0.62 (0.19–1.14)	0.809
TNF-α <br>(pg/mL)	1.85 (0.61–2.67)	0.99 (0.51–2.16)	0.297
IL-10 <br>(pg/mL)	1.27 (0.84–3.86)	1.27 (0.74–1.64)	0.308

Analysis of immune status indicators, including the IFN-γ/IL-4 ratio, TNF-α/IL-4 ratio, and TNF-α/IL- 10 ratio, revealed a trend toward lower IFN-γ/IL-4 ratios in the SAI group. However, this difference did not reach statistical significance (Table 6).

**Table 6:** Comparison of Immune Status Indicators Between SAI and nSAI Groups.

Immune Status <br>Indicator	SAI Group	nSAI Group	P Value
TNF-α/IL-4	2.43 <br>(0.52–4.9)	1.99 <br>(1.01–5.33)	0.725
TNF-α/IL-10	0.61 <br>(0.21–2.69)	0.88 <br>(0.3–3.2)	0.506
IFN-γ/IL-4	0.94 <br>(0.18–1.74)	1.25 <br>(0.61–4.06)	0.097

## Discussion

Stroke is a common cerebrovascular disease characterized by irreversible brain tissue damage and complex pathological mechanisms, including excitotoxicity caused by glutamate, calcium overload, oxida tive stress, and inflammatory injury. Inflam mation mediated by the immune system plays a crucial role in post-stroke brain tissue damage [13] [14]. Immune status influences the local pathophysiological changes and outcomes in ischemic brain tissue. Proinflammatory states exacerbate local damage, whereas anti-inflammatory states protect brain tissue, promoting the clearance of necrotic cells and preventing further injury. The intricate connection between central nervous system inflammation and systemic immunity underscores the systemic nature of the human body. For example, following stroke, the breakdown of the blood-brain barrier (BBB) allows peripheral immune cells to infiltrate the brain. These infiltrating Th cells secrete various cytokines and interact with microglia, modulating their polarization state and collectively influencing the extent of local brain tissue damage and recovery [14] [15].

The relationship between the nervous and immune systems is bidirectional. The immune system not only influences the pathophysiological processes of stroke but is also affected by stroke events. For instance, post-stroke immunosuppression may occur, the mechanisms of which remain unclear. The hypothalamic- pituitary-adrenal axis and the sympathetic nervous system may play roles in this process, serving as protective mechanisms to suppress central inflammation and regulate peripheral immune status [16]. However, excessive immunosuppression can have adverse effects, increasing the risk of post-stroke infections, which further impact hospitalization duration and short-term mortality in stroke patients [17] [18].

Given the importance of immune factors in stroke progression, research into immune mechanisms has garnered increasing attention. Multiple factors influence immune status, and some of these factors can be artificially controlled, making immune mechanisms in stroke progression potential clinical therapeutic targets [19] [20]. In this study, serum levels of cytokines, including IL-2, IL-6, TNF-α, IFN-γ, IL-4, IL-10, and IL-17A, were measured within 72 hours of stroke onset. Immune status indicators, such as IFN-γ/IL-4, TNF-α/IL-4, and TNF-α/IL-10 ratios, were calculated. Laboratory test results, imaging data, and patient history were collected to analyze factors influencing peripheral immunity and to provide clinical data for investigating immune mechanisms in acute ischemic stroke.

Homocysteine is a recognized risk factor for cardiovascular and cerebrovascular diseases, participating in and accelerating atherosclerosis progression [21] [22]. In addition, homocysteine exhibits immunomodulatory effects, potentially influencing T cell activation and polarization processes. Previous studies have demonstrated that elevated homocysteine levels promote the secretion of cytokines such as IL-2, IFN-γ, and IL-10, while IL-4 and IL-5 levels remain unaffected. Exogenous homocysteine has also been shown to induce Th cell polarization toward the Th17 phenotype. However, these studies were primarily conducted in vitro or in animal models, with limited clinical research on the impact of homocysteine on cytokines in acute ischemic stroke patients.

Our results indicate that serum levels of IFN-γ, IL-10, and IL-6 were significantly higher in the high-homocysteine group (P < 0.05). Levels of IL-17A, IL- 2, and the IFN-γ/IL-4 ratio showed increasing trends but did not reach statistical significance. IFN-γ and IL- 6 are pro-inflammatory cytokines. IFN-γ, secreted by Th1 cells, mediates macrophage activation and promotes M1 polarization of microglia, shifting immune status toward a pro-inflammatory state and exacerbating brain inflammation. IL-6 is rapidly produced following infection and tissue injury and plays a pathological role in chronic inflammation and autoimmunity. IL-10, an anti-inflammatory cytokine, inhibits the expression and activation of cytokine receptors, antagonizing inflammation by suppressing the expression of pro-inflammatory mediators, including IL-1β, TNF-α, and IFN-γ, thereby protecting neural tissue from further inflammatory damage during ischemic injury. IL-17A and IL-2 are also pro-inflammatory cytokines. The IFN-γ/IL-4 ratio reflects the balance between Th1 and Th2 subpopulations, with higher ratios indicating a predominance of Th1. Our findings align with previous in vitro and animal studies, showing that elevated homocysteine increases Th1-associated cytokines (e.g., IFN-γ) and proinflammatory cytokines (e.g., IL-6). Additionally, our results reveal that anti-inflammatory cytokine IL-10 was elevated in the high-homocysteine group, though the underlying mechanisms remain unclear. The upward trends in IL-17A, IL-2, and the IFN-γ/IL-4 ratio suggest that hyperhomocysteinemia may shift immune status toward a pro-inflammatory direction.

Immunosuppression commonly occurs during the acute phase of ischemic stroke, possibly as a self-protective mechanism to mitigate damage from excessive inflammation. However, systemic immune changes and local brain inflammation may occur concurrently. Excessive immunosuppression can lead to adverse outcomes, such as post-stroke infections. Th1 cells exacerbate focal brain inflammation through their pro-inflammatory mediators and by promoting the pro-inflammatory polarization of microglia. Conversely, Th2 cells counteract Th1-mediated effects, exerting anti-inflammatory and neuroprotective roles. However, a shift toward Th2-dominant antiinflammatory responses has been shown to increase the risk of post-stroke pneumonia in experimental models. Recombinant IL-33 promotes Th2 responses following focal ischemic stroke, reducing infarct size by elevating plasma Th2 cytokine levels and decreasing pro-inflammatory microglia and macrophages in brain tissue. Nonetheless, IL-33-treated mice exhibited increased bacterial lung infections and functional deficits post-stroke, with higher 24-hour mortality rates.

This study analyzed the immune status of stroke patients with and without infections, including cytokine levels and immune status indicators such as the IFN-γ/IL-4 ratio. Results showed significantly higher IL-4 levels in the SAI group compared to the nSAI group. The lower IFN-γ/IL-4 ratio in the SAI group, although not statistically significant (P = 0.059), suggests more pronounced immunosuppression. Patients included in the study had moderate stroke severity (NIHSS scores between 4 and 13), and no differences in overall NIHSS scores were observed between SAI and nSAI groups. This finding indicates that the severity of immunosuppression, rather than stroke severity, may underlie the risk of post-stroke infections.

Homocysteine-lowering therapies, such as folate and vitamin B12 supplementation, have shown promise in reducing homocysteine levels and modulating immune responses in various clinical settings. By decreasing pro-inflammatory cytokine production and restoring immune balance, these interventions may help mitigate post-stroke inflammatory responses and reduce the risk of stroke-associated infections. Additionally, folate and B12 supplementation have been linked to improved endothelial function and neuroprotection, which may contribute to better functional recovery in stroke patients. Future studies should explore the direct impact of these therapies on immune modulation in acute ischemic stroke to validate their clinical benefits.

## Conclusion

This study highlights the critical role of immune responses in the progression and outcomes of ischemic stroke. Immune dysregulation, influenced by factors such as serum homocysteine levels, significantly affects cytokine profiles and post-stroke events. Higher homocysteine levels were associated with elevated pro-inflammatory cytokines, including IFN-γ and IL-6, as well as the anti-inflammatory cytokine IL- 10, suggesting a complex immune modulation during the acute phase of stroke. Our findings indicate that immune status strongly influences the risk of poststroke infections. Patients exhibiting a shift toward anti-inflammatory immune responses (e.g., higher IL- 4 levels and lower IFN-γ/IL-4 ratios) were more susceptible to infections, irrespective of stroke severity. This underscores the dual role of immune responses in mitigating central inflammation while predisposing to systemic immunosuppression. The study provides valuable clinical data on the interplay between immune mechanisms and stroke pathophysiology. Future research should further explore immunomodulatory strategies targeting cytokine dynamics to improve stroke management and reduce adverse outcomes.

## Dodatak

### Conflict of interest statement

All the authors declare that they have no conflict of interest in this work.
